# Hepatocyte SREBP signaling mediates clock communication within the liver

**DOI:** 10.1172/JCI163018

**Published:** 2023-04-17

**Authors:** Dongyin Guan, Hosung Bae, Dishu Zhou, Ying Chen, Chunjie Jiang, Cam Mong La, Yang Xiao, Kun Zhu, Wenxiang Hu, Trang Minh Trinh, Panpan Liu, Ying Xiong, Bishuang Cai, Cholsoon Jang, Mitchell A. Lazar

**Affiliations:** 1 Division of Diabetes, Endocrinology, and Metabolism, Department of Medicine, Baylor College of Medicine, Houston, Texas, USA.; 2 Department of Biological Chemistry, University of California Irvine, Irvine, California, USA.; 3 Institute for Diabetes, Obesity, and Metabolism, Perelman School of Medicine at the University of Pennsylvania, Philadelphia, Pennsylvania, USA.; 4 Department of Basic Research, Guangzhou Laboratory, Guangdong, China.; 5 Division of Liver Diseases, Department of Medicine, Icahn School of Medicine at Mount Sinai, New York, New York, USA.; 6 Division of Endocrinology, Diabetes, and Metabolism, Department of Medicine and; 7 Department of Genetics, the University of Pennsylvania Perelman School of Medicine, Philadelphia, Pennsylvania, USA.

**Keywords:** Hepatology, Metabolism, Epigenetics, Homeostasis

## Abstract

Rhythmic intraorgan communication coordinates environmental signals and the cell-intrinsic clock to maintain organ homeostasis. Hepatocyte-specific KO of core components of the molecular clock *Rev-erb*α and -β (*Reverb*-hDKO) alters cholesterol and lipid metabolism in hepatocytes as well as rhythmic gene expression in nonparenchymal cells (NPCs) of the liver. Here, we report that in fatty liver caused by diet-induced obesity (DIO), hepatocyte SREBP cleavage–activating protein (SCAP) was required for *Reverb-*hDKO–induced diurnal rhythmic remodeling and epigenomic reprogramming in liver macrophages (LMs). Integrative analyses of isolated hepatocytes and LMs revealed that SCAP-dependent lipidomic changes in REV-ERB–depleted hepatocytes led to the enhancement of LM metabolic rhythms. Hepatocytic loss of REV-ERBα and β (REV-ERBs) also attenuated LM rhythms via SCAP-independent polypeptide secretion. These results shed light on the signaling mechanisms by which hepatocytes regulate diurnal rhythms in NPCs in fatty liver disease caused by DIO.

## Graphical abstract

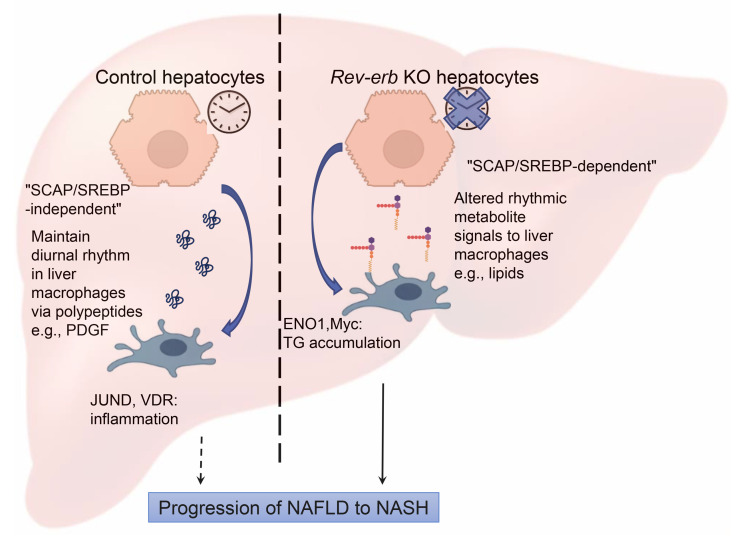

## Introduction

In almost all organisms, circadian rhythms, which regulate most biological processes and have strong ties to human health and disease, anticipate and adapt to daily environmental changes (1–3). The mammalian circadian clock system consists of a master clock in the suprachiasmatic nucleus that hierarchically controls circadian rhythms in peripheral tissues (4), where a large percentage of the protein-coding transcriptome exhibits daily rhythmic expression in rodents (5) as well as in nonhuman primates (6). Recent advances in circadian biology indicate diurnal rhythmic communication between different cells within the liver and coordinating regulation of rhythmic gene expression (7, 8). However, the underlying mechanisms remain largely unclear, and it is unknown whether this occurs during pathological conditions such as fatty liver disease.

It is generally appreciated that the core components of the molecular clock, including BMAL1, CLOCK, REV-ERB, CRY, and PER, form interlocking feedback loops to regulate the rhythmic expression of clock output genes (2, 9). Deletion of core clock components induces circadian remodeling without complete loss of rhythmicity (7, 10), suggesting that other pathways contribute to the generation and maintenance of biological rhythms. Indeed, diet-induced obesity (DIO) causes massive diurnal rhythmic transcriptomic remodeling with mild changes in the expression of core clock components (11, 12), further indicating that additional factors contribute to rhythmic gene expression. Whether these noncanonical pathways control secreted polypeptides or metabolites (13, 14) that may non-cell-autonomously influence rhythmic gene expression within an organ is unknown.

Here, we found that lipid and cholesterol metabolism–related gene expression was altered in hepatocytes upon the disruption of core clock component REV-ERBs in diet-induced fatty liver disease. Specific loss of REV-ERBs in hepatocytes (*Reverb*-hDKO) had profound effects on gene expression in nonparenchymal cells (NPCs), suggesting intraorgan communication. SREBP, a master regulator of lipid and cholesterol metabolism (15), was altered in hepatocytes lacking REV-ERBs, and hepatocyte disruption of SREBP signaling by deletion of SREBP cleavage–activating protein (SCAP) restored the *Reverb*-hDKO–induced rhythmic expression of genes in the liver macrophages (LMs), including resident Kupffer cells (KCs) and monocyte-derived macrophages (MoMs). Ligand receptor analysis in hepatocytes and LMs revealed hepatocyte SCAP-controlled polypeptide ligands and lipids that contribute to intraogran clock communication.

## Results

### SREBP signaling is required for communication from hepatocytes lacking REV-ERBs to NPCs of the liver.

**Figure 1:**
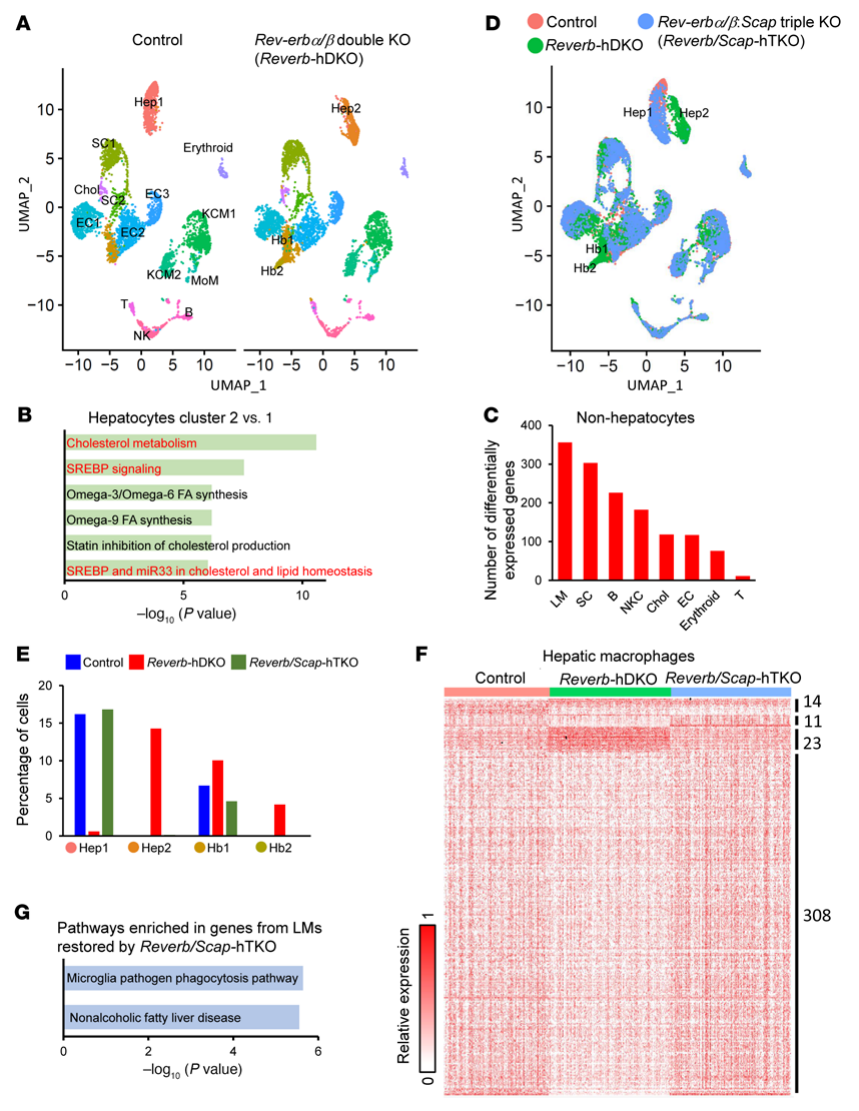
SREBP signaling is required for intraorgan communication from hepatocytes lacking REV-ERBs. (**A**) UMAP visualization of cell clusters in livers from control and *Reverb*-hDKO mice fed a HFHSD for 4 weeks. (**B**) KEGG pathways enriched in the genes differentially expressed between hepatocyte cluster 2 versus cluster 1. (**C**) Number of DEGs in nonhepatocytes upon *Reverb* hDKO. (**D**) UMAP visualization of the overlapping cell cluster in livers from control, *Reverb*-hDKO, and *Reverb/Scap*-hTKO mice fed a HFHSD for 4 weeks. (**E**) Percentages of the indicated cell populations. (**F** and **G**) Heatmap of (**F**) and pathways enriched in (**G**) genes from LMs restored by *Reverb/Scap* hTKO. Hep, hepatocyte; Hb, hepatoblast; SC, stellate cell; EC, endothelial cell; B, B cell; T, T cell; erythroid, erythroid cell; Chol, cholangiocyte.

Hepatocytes regulate diurnal rhythms in NPCs under normal conditions (7), but it is unknown whether this occurs in a pathological condition such as fatty liver disease. Control and *Reverb*-hDKO mice were put on an obesogenic diet that causes fatty liver (11), and single-cell RNA-Seq identified populations of hepatocytes, macrophages (including resident KCs with M1 and M2 status, and MoMs), stellate cells, endothelial cells, and immune cells on the basis of cell-specific markers (16, 17) in control and *Reverb*-hDKO livers (Figure 1A and Supplemental Figure 1A; supplemental material available online with this article; https://doi.org/10.1172/JCI163018DS1). We observed both mature and less-well-differentiated hepatocytes (hepatoblasts) in control and *Reverb*-hDKO livers. We noted the greatest differences in hepatocytes, with SREBP signaling and related cholesterol and lipid metabolic pathways enriched in *Reverb*-hDKO hepatocytes (Figure 1, A and B). Hepatocyte *Reverb*-hDKO also altered gene expression in NPCs, with macrophages being the most affected cell type (Figure 1C). Regarding hepatic stellate cells, the second most affected cell type, we found that 303 genes were differentially expressed upon DKO, in which genes for hepatocyte growth factor receptor– (c-Met–) and c-Myc–related pathways were enriched (Figure 1C and Supplemental Figure 1B). These 2 pathways are related to the activation of hepatic stellate cells (18, 19).

To test the role of SREBP signaling from *Reverb*-hDKO hepatocytes to NPCs, we generated mice with hepatocyte-specific KO of both REV-ERBs as well SCAP (*Reverb/Scap*-hTKO). Remarkably, loss of SCAP restored hepatocyte and hepatoblast population changes due to *Reverb*-hDKO (Figure 1, D and E, and Supplemental Figure 1, C and D). Moreover, 93% (331 of 356) of differentially expressed genes (DEGs) in LMs from *Reverb*-hDKO livers were restored in the *Reverb/Scap*-hTKO LMs (Figure 1F). The restored genes were enriched for pathways mediating phagocytosis and nonalcoholic fatty liver disease (Figure 1G). Moreover, the MoMs, but not resident KCs (KCM1 and KCM2), were increased in the *Reverb*-hDKO livers and could be rescued by further SCAP KO (Supplemental Figure 1C), suggesting that the restored gene expression levels were not due to the changes in the cell population. While the body weights of mice were increased by the obesogenic diet, there was no significant difference across different genetic models, indicating that the increased number of MoMs and activation of stellate cells were earlier events than the changes in body weight (Supplemental Figure 1, C and E). These findings support the role of hepatocyte SREBP signaling as an effector of hepatocyte REV-ERB communication to NPCs in the fatty liver.

### SREBP signaling is required for the rhythmic transcriptomic remodeling in LMs upon Reverb-hDKO.

**Figure 2:**
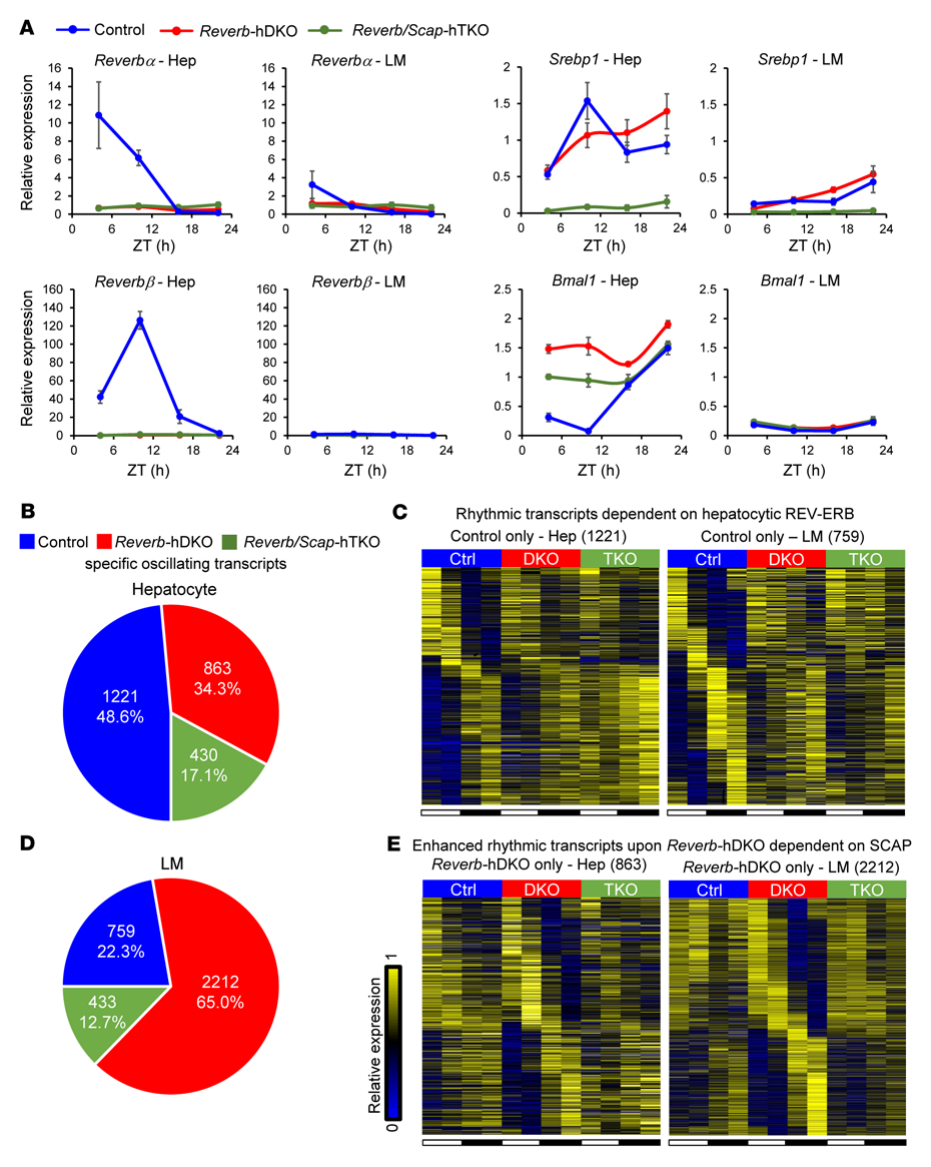
SREBP signaling is required for rhythmic transcriptomic remodeling in LMs upon loss of hepatocytic REV-ERBs. (**A**) Relative mRNA expression of the indicated genes in livers from *Reverb*-hKO, and *Reverb/Scap*-hTKO mice fed a HFHSD for 4 weeks. The gene expression data are expressed as the mean ± SEM (*n* = 4–6 per group). (**B**) Number and percentage of control-, *Reverb*-hDKO–, and *Reverb/Scap*-hTKO–specific oscillating transcripts in hepatocytes. (**C**) Heatmap of rhythmic transcripts in hepatocytes or LMs dependent on hepatocytic REV-ERB. (**D**) Number and percentage of control-, *Reverb*-hDKO–, and *Reverb/Scap*-hTKO–specific oscillating transcripts in LMs. (**E**) Heatmap of enhanced rhythmic transcripts upon SCAP-dependent *Reverb* hDKO.

To determine the impact of the hepatocyte clock on diurnal rhythms in LMs and the role of SREBP in this regulation, we measured rhythmic gene expression profiles of hepatocytes and LMs in control, *Reverb*-hDKO, and *Reverb/Scap*-hTKO fatty livers. The expression levels of *Reverb*α and *Reverb*β were markedly reduced in hepatocytes, as expected, and consistent with this, the major REV-ERB target gene *Bmal1* was markedly derepressed in the *Reverb*-hDKO and *Reverb/Scap*-hTKO LMs, whereas *Srebp1* was reduced only in *Reverb/Scap*-hTKO livers (Figure 2A). Compared with hepatocytes, expression levels of the clock genes *Reverb*α, *Reverb*β, and *Bmal1* were lower in LMs, as expected (7), and only mildly altered by *Reverb*-hDKO and *Reverb/Scap*-hTKO (Figure 2A). Moreover, consistent with the gene expression changes suggesting altered SREBP signaling, triglycerides (TGs) were increased in *Reverb*-hDKO livers but rescued by further KO of *Scap* (Supplemental Figure 2A).

In hepatocytes, 1,221 transcripts that were rhythmic in control livers exhibited lost or attenuated diurnal rhythms in the *Reverb*-hDKO livers (Figure 2, B and C, and Supplemental Table 1A), which were highly enriched for circadian rhythm–related genes (Supplemental Figure 2B). Interestingly, 863 transcripts had increased or amplified rhythms in *Reverb*-hDKO livers (Figure 2, B and E, and Supplemental Table 1B), and these rhythmic transcript gains were notably related to fatty liver disease (Supplemental Figure 2C). Similar results were observed in *Reverb*-hDKO livers from mice on a normal chow diet (7), and the present results demonstrate that the phenomenon of gained rhythmicity upon disruption of the hepatocyte clock also occurred in mice with fatty livers. Loss of SCAP had little effect on the genes that lost rhythmicity in the *Reverb*-hDKO livers (Figure 2C), consistent with direct regulation of these genes by REV-ERBs. Remarkably, however, simultaneous deletion of hepatocyte SCAP reversed the majority of gained rhythms (Figure 2E).

SCAP KO also ablates the expression of *Srebp2* (20), and, indeed, we found that both *Srebp2* and its classic target gene *Hmgcr* were markedly reduced with attenuated rhythmicity in *Reverb*-hDKO livers (Supplemental Figure 2F). These results strongly suggest that SREBF1 had a more prominent role in the hepatocyte genes that gained rhythm in the *Reverb*-hDKO livers than in those that lost rhythmicity, consistent with the notion that the gained rhythms were secondary to rhythms of SREBP that were amplified in hepatocytes lacking REV-ERBs (7). The rhythm of hepatocytic *Srebp1* in the control liver is likely explained by the rhythm of *Insig2*, which blocks the transcription of *Srebp1* (21) and is directly transcriptionally repressed by REV-ERBα, based on the ChIP-Seq analysis (Supplemental Figure 2, F and G). The phase-shifted rhythm of *Srebp1* in *Reverb*-hDKO liver was also previously observed in livers of chow-fed mice (7). The etiology is unclear but may reflect signals from the central clock or the environment, since the rhythm can be entrained by reversed-phase feeding in chow-fed mice (7).

We also identified LM genes whose rhythms were affected by the loss of REV-ERBs in hepatocytes, with 759 lost or attenuated rhythmic genes and 2,212 genes with gained or amplified rhythms (Figure 2, C–E, Supplemental Table 1D, and Supplemental Table 1C). Remarkably, the vitamin D receptor (VDR) signaling pathway, whose activation in LMs ameliorates liver inflammation and steatosis (22, 23), was enriched in the rhythmicity-lost genes (Supplemental Figure 2B). The rhythmicity-gained genes were related to inflammatory responses (Supplemental Figure 2C), indicating that this rhythmic remodeling in LMs was a maladaptive response to the *Reverb*-hDKO. Simultaneous ablation of SCAP/SREBP signaling in hepatocytes had little effect on the loss of gene rhythmicity in LMs (Figure 2C), but reversed changes in the majority of the LMs genes that gained rhythmicity upon loss of REV-ERBs in hepatocytes (Figure 2E). Overall, 22.3% of rhythmic transcripts in LMs were dominantly regulated by hepatocyte REV-ERBs, while *Reverb/Scap*-hTKO restored 65% of enhanced rhythmic transcripts upon *Reverb*-hDKO. Of note, although the *Reverb*-hDKO caused massive rhythmic remodeling, the rhythmic transcripts in hepatocytes and LMs from control and *Reverb*-hDKO livers were enriched in similar phases (ZT15 in control-specific rhythms, ZT3 and ZT18 in *Reverb*-hDKO–specific rhythms) (Supplemental Figure 2, D and E).

Given the importance of SCAP in liver lipid metabolism and previous evidence that SREBP1 and its target genes gain diurnal rhythmicity in DIO mice that is dependent on hepatocyte SCAP expression (11), we further sought to determine the role of SCAP in hepatocytes and macrophages. Core clock genes retained their rhythmic expression in hepatocyte-specific *Scap* KO (*Scap*-hKO) hepatocytes from mice fed a 4-week high-fat, high-sucrose diet (HFHSD) (Supplemental Figure 3A). Indeed, the enhanced, but not disrupted, rhythmic transcripts remodeled by DIO were hepatocyte SCAP dependent (Supplemental Figure 3B and Supplemental Table 2). We also determined the specific requirement of SCAP/SREBP signaling in the REV-ERB–dependent changes in rhythmic gene expression by comparing changes in diurnal gene expression that occurred in the hepatocyte SCAP–KO livers with those seen in the *Reverb/Scap-*hTKO livers. Importantly, a majority of the rhythmic changes in hepatocytes and LMs that were noted in the *Reverb-*DKO livers and reversed in the *Reverb/Scap-*hTKO livers were not altered in the livers of mice lacking hepatocytic SCAP, indicating a role for SCAP/SREBP in specifically offsetting changes due to the loss of REV-ERBs (Supplemental Figure 3C).

### Ligands from hepatocytes reprogram REV-ERB–dependent oscillating enhancers in LMs.

**Figure 3:**
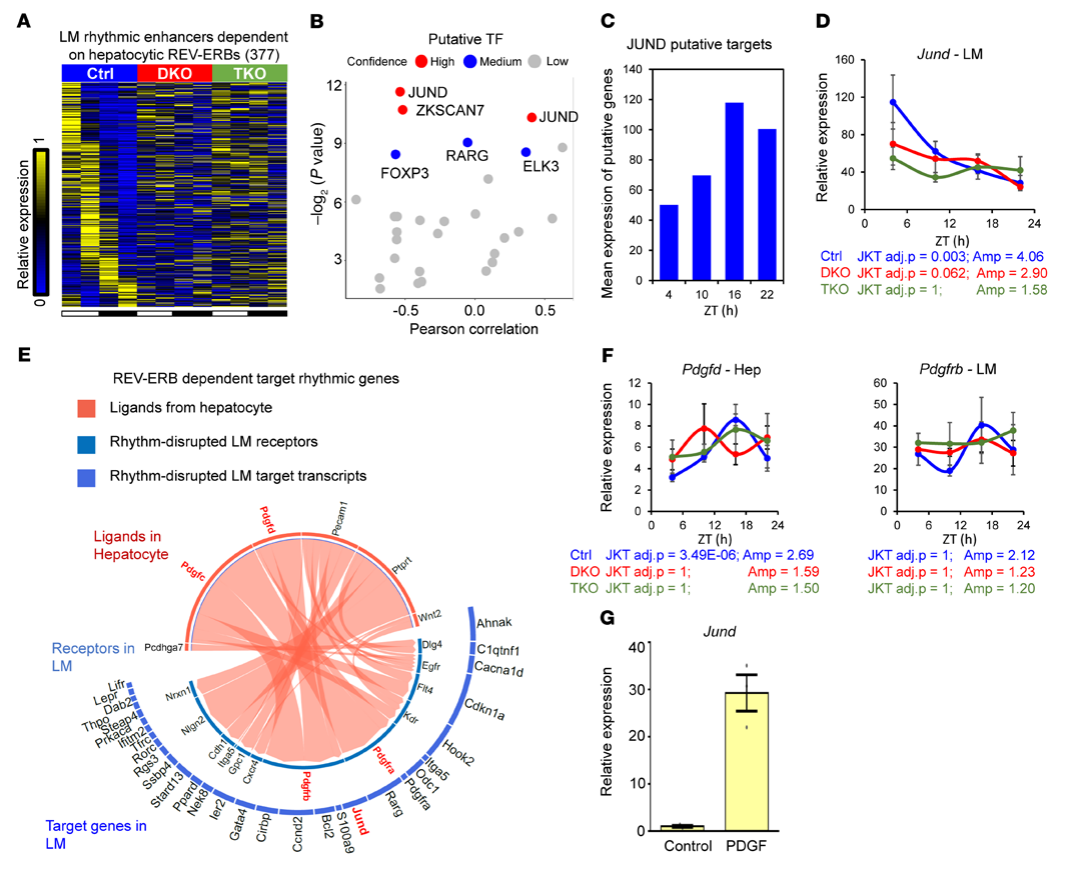
Ligands from hepatocytes reprogram REV-ERB–dependent oscillating enhancers in LMs. (**A** and **B**) Heatmap (**A**) and IMAGE analysis (**B**) of rhythmic enhancers in LMs dependent on hepatocytic REV-ERBs. (**C**) Mean expression of putative target genes of JUND in isolated LMs from control liver. (**D**) Relative expression of *Jund* in LMs of livers from control, *Reverb*-hDKO, and *Reverb/Scap*-hTKO mice fed a HFHSD for 4 weeks. Data are presented as the mean ± SEM (*n* = 3 per time point). (**E**) Ligand-receptor pair analysis. Circle plots show links between the predicted ligands from hepatocytes (red) with their associated receptors from LMs (blue) associated with rhythmic transcripts in LMs dependent on hepatocytic REV-ERB potentially targeted by the ligand-receptor pairs. (**F**) Relative expression of *Pdgfd* in hepatocytes and *Pdgfrb* in LMs. Data are presented as the mean ± SEM (*n* = 3 per time point). (**G**) Relative expression of *Jund* in immortalized KCs with BSA (control) or PDGF treatment. Data are presented as the mean ± SEM (*n* = 3 per time point). The gene expression data in **D**, **F**, and **G** are expressed as the mean ± SEM (*n* = 4–6 per group). Amp, amplitude; adj.p, adjusted *P* value; Ctrl, control.

To uncover transcription factors (TFs) responsible for transcriptome remodeling in *Reverb*-hDKO livers, we mapped enhancer RNA (eRNA) expression in isolated LMs by mapping RNA-Seq reads to intergenic regions of open chromatin determined by assay for transpose-accessible chromatin using sequencing (ATAC-Seq) (24). We then defined LM enhancers that functioned predominantly in control LMs (REV-ERB–dependent) and *Reverb*-hDKO only (SCAP-dependent remodeling upon *Reverb*-hDKO) (Figure 3A, Supplemental Figure 4A, and Supplemental Table 3, A and B).

Integrated analysis of motif activity and gene expression (IMAGE) (25) identified putative TFs in response to the signals from hepatocytes regulating the rhythmic gene expression in LMs. For example, JUND, a member of the AP1 family that activates and represses transcription depending on its associated proteins (26), was identified as a TF that was responsible for hepatocyte REV-ERB–dependent rhythmic transcripts (Figure 3, B and C). Indeed, the expression of *Jund* in LMs was rhythmic and had a higher amplitude in control than in *Reverb*-hDKO and *Reverb/Scap*-hTKO livers (Figure 3D). A short isoform of enolase 1 (ENO1, also known as Myc promoter–binding protein 1), which binds to the c-Myc promoter and acts as a TF (27), was identified as a TF that potentially regulated SCAP-dependent rhythmic remodeled transcripts (Supplemental Figure 4, B and C), and, consistent with this prediction, *Eno1* was rhythmic and had higher amplitude in LMs from *Reverb*-hDKO than in control and *Reverb/Scap*-hTKO livers (Supplemental Figure 4D).

To explore potential signals from hepatocytes to LMs, we used NicheNet (28) to identify ligands that were altered in hepatocytes and receptors and putative target genes whose expression in LMs was dependent on hepatocyte REV-ERBs (Figure 3E and Supplemental Figure 4E). In this way, platelet-derived growth factor D (PDGFD), a major growth factor family involved in macrophage polarization and cell proliferation (29), was identified as a regulatory ligand from hepatocytes, with rhythmic expression in control but not in *Reverb*-hDKO and *Reverb/Scap*-hTKO livers (Figure 3F). We further validated the protein levels of PDGFD in control and *Reverb*-hDKO livers (Supplemental Figure 4F). The PDGF receptor *Pdgfrb* was expressed in LMs, although it was not statistically rhythmic in all 3 genetic models (Figure 3F). Interestingly, *Jund* is a known target gene of the PDGFD-PDGFRB ligand-receptor pair (30), and, indeed, treatment of immortalized KCs with PDGF increased the expression levels of *Jund* (Figure 3G). This result was consistent with the notion that rhythmically increased PDGF from hepatocytes lacking REV-ERBs mediates the remodeling of oscillating enhancers and transcription in LMs by inducing JUND. JUND is also required for VDR-mediated transcription activation (31, 32), in which VDR target genes are enriched in LM rhythmic transcripts dependent on hepatocytic REV-ERBs (Figure 2B).

We also identified *Bmp5*, *Inhba*, and *Tnfsf12* as ligands from hepatocytes that regulated enhanced rhythmic transcripts upon *Reverb*-hDKO dependent on SCAP (Supplemental Figure 4E). For example, the rhythmicity and amplitude of both ligand *Bmp5,* which may be involved in LM differentiation (28), in hepatocytes and receptor *Bmpr1a* in LMs were enhanced in *Reverb*-hDKO livers compared with control and *Reverb/Scap*-hTKO livers (Supplemental Figure 4G). To further explore the transcriptional mechanism underlying these enhanced rhythmic ligands in LM, we used CistromeDB (33) to perform TF similarity screening based on all published macrophage cistromes. Consistent with IMAGE analysis, SP11 and Myc, which are regulated by ENO1, are the top regulatory TFs (Supplemental Figure 4H). However, the regulatory TFs identified from IMAGE analysis and TF similarity screening (Supplemental Figure 4, B and H) were not the top target transcripts in this ligand-receptor analysis. Moreover, REV-ERBα only bound the promoter region of 31% of the SCAP-dependent enhanced rhythmic ligands in hepatocytes, while the promoters of 58% of the disrupted rhythmic ligands upon *Reverb*-hDKO were occupied by REV-ERBα (Supplemental Figure 4I). These results suggest that additional mechanisms and signals, such as lipids and metabolites, could mediate the rhythmicity of these SCAP-dependent transcripts.

### Hepatocyte lipid metabolism regulates SCAP-dependent diurnal rhythms in LMs upon REV-ERB disruption.

**Figure 4:**
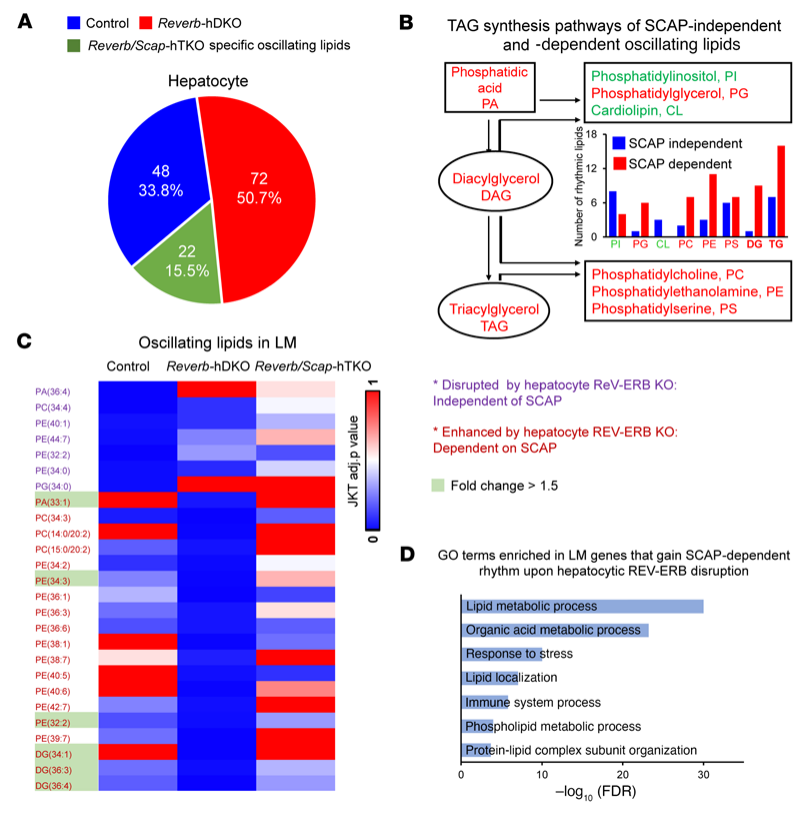
LM diurnal rhythms controlled by SCAP-dependent lipid metabolism in REV-ERB–depleted hepatocytes. (**A**) Number and percentage of control-, *Reverb*-hDKO–, and *Reverb/Scap*h-DTKO–specific oscillating lipids. (**B**) TAG synthesis pathways of SCAP-dependent oscillating lipids whose species number was increased (red) or decreased (green) upon REV-ERB disruption. PI, phosphatidylinositol. (**C**) Heatmap of rhythmic lipids in LMs upon hepatocytic REV-ERB disruption independent of or dependent on SCAP. The rhythms of SCAP-independent lipids upon hepatocytic REV-ERB disruption are labeled in purple, and SCAP-dependent lipids are labeled in violet. The lipids whose abundance was increased upon hepatocytic REV-ERB disruption are highlighted in a light-green background. (**D**) GO terms enriched in gained rhythmic genes in LMs upon SCAP-dependent hepatocytic REV-ERB disruption.

Given the central role of SCAP/SREBP signaling in lipid metabolism, we focused on lipid metabolites whose rhythms were dysregulated in the absence of hepatocyte REV-ERBs. Upon deletion of REV-ERBs in hepatocytes, 48 lipid species lost or attenuated rhythms, while 80 lipid species gained rhythmicity (Figure 4A and Supplemental Table 4A). Remarkably, 72 of 80 of the gained rhythms were dependent on SCAP/SREBP signaling (Figure 4A and Supplemental Figure 5).

Consistent with the regulatory role of SREBP/SCAP in lipid metabolism, the rhythmicities of precursors of major phospholipids, including phosphatidylglycerol (PG), phosphatidylcholine (PC), phosphatidylethanolamine (PE), phosphatidylserine (PS), diacylglycerol (DAG), and triacylglycerol (TAG) (34), whose activities are reportedly involved in nonalcoholic fatty liver disease, were enriched in the SCAP-dependent group (Figure 4B). To determine whether the rhythmic remodeling of these lipids in hepatocytes lacking REV-ERBs altered the diurnal rhythm of LMs, we determined the lipid profiles of LMs isolated from control, *Reverb*-hDKO, and *Reverb/Scap*-hDKO mice. Interestingly, there were more lipid species, including PC, PE, and DAG, oscillating in LMs isolated from *Reverb/Scap*-hDKO mice (Figure 4C and Supplemental Table 4B). Indeed, hundreds of genes that became rhythmic in LMs in a SCAP-dependent manner upon hepatocytic REV-ERB disruption are known to be involved in lipid metabolism (35, 36) (Figure 4D). These results suggest that SCAP-dependent hepatocyte lipid metabolism alters diurnal rhythms in LMs upon REV-ERB disruption.

## Discussion

Each cell type exhibits a distinct diurnal rhythm that is linked to the corresponding functions of that cell type in specific pathophysiological contexts (37, 38). Plasma samples collected from humans and rodents at different times of day activate a luciferase reporter system in a diurnal manner, with a nearly inverted phase in culture cells, indicating a regulatory role of systemic blood-borne oscillating signals in rhythmic gene expression in peripheral cell types (39). However, the local signaling molecules and the mechanisms by which they drive the cell-type–specific diurnal rhythm in pathological contexts are largely unknown. Our results demonstrate that, in the fatty liver, loss of core clock component REV-ERBs in hepatocytes led to altered rhythmic gene expression in NPCs, with LMs being the most affected cell type upon REV-ERB disruption.

Immune responses in the liver exhibit diurnal variation, for example in response to LPS or in the context of nonalcoholic steatohepatitis (NASH) (40, 41). Previous studies have identified regulatory roles of core clock genes, including RORα (41) and REV-ERBs (42), in the diurnal rhythms of LMs. Here, on the basis of enhancer mapping and motif analysis, we identified noncanonical clock regulators, including JUND and ENO1, in rhythmic LM gene expression. For example, the gain of ENO1 rhythmicity that is dependent on hepatocytic SCAP may reflect c-Myc activity (as ENO1/MBP1 can regulate *Myc* transcription) and TGF-β signaling, which may signal the initiation of a gene program that activates profibrotic stellate cells (43, 44). These newly identified clock regulators could be targeted at a specific time of day according to their rhythmicity to optimize treatment of the progression of nonalcoholic fatty liver disease (NAFLD) or NASH.

Hepatocyte communication of rhythm signals to LMs is largely dependent on hepatocytic SCAP, which is required for the processing and stability of SREBP, the master transcriptional regulator of liver lipid metabolism (45, 46). LMs are critical in the progression from fatty liver to NASH (47–49), and, indeed, SCAP-dependent changes in hepatocytic lipid metabolism play a crucial role in producing gained rhythms of genes related to inflammation in KCs. Loss of hepatocytic REV-ERBs also leads to disruption of LM rhythms, in part by SCAP/SREBP-independent regulation of PDGF, which regulates LM genes involved in vitamin D signaling pathways implicated in fatty liver and NASH (22, 50).

Taken together, these findings demonstrate the communication of local metabolic and rhythmic protein signals from hepatocytes to NPCs, which is altered in fatty liver. Similar clock-dependent intraorgan communication pathways are likely to exist in other peripheral tissues and to be disrupted under pathological conditions.

## Methods

### Animal studies.

*Rev-erb*α*^fl/fl^* *Rev-erb*β*^fl/fl^* animals were generated as previously described (7). *Rev-erb*α*^fl/fl^* *Rev-erb*β*^fl/fl^* *Scap^fl/fl^* animals were generated by breeding the *Rev-erb*α*^fl/fl^* *Rev-erb*β*^fl/fl^* mice with *Scap^fl/fl^* mice on a C57BL/6 background. For specific deletion of *Rev-erb*α*/*β or *Rev-erb*α*/*β *Scap* in adult hepatocytes, adeno-associated viruses (AAVs) encoding GFP or CRE driven by the hepatocyte-specific TBG promoter (AAV-TBG-GFP for control and AAV-TBG-CRE for KO) were prepared by the UPenn Vector Core and were intravenously injected into 8-week-old mice at 1.5 × 10^11^ genome copies (GCs) per mouse. After injection, the mice were fed a HFHS diet (Research Diet, catalog D12492) for 4 weeks. All mice were housed under a 12-hour light/12-hour dark cycle, with lights on at 7 am (zeitgeber time 0, ZT0) and lights off at 7 pm (ZT12).

### Single-cell RNA-Seq.

Single-cell RNA-Seq and cell isolation were performed as described previously with minor modifications (51, 52). In brief, mice were anesthetized, and the livers were perfused with calcium-free HBSS containing 0.2 mg/mL EDTA, followed by sequential digestion with HBSS with 0.5 mg/mL collagenase type II, 0.04 mg/mL trypsin inhibitor, and 1.37 mM calcium for 3 minutes at 37°C through the portal vein. The liver was dissociated and the cell suspension was centrifuged at 30*g* for 5 minutes. The NPCs were enriched in the suspension and washed with MACS buffer (1× PBS, pH 7.2, 0.5% BSA, and 2 mM EDTA). A total of 16,000 cells were immediately processed with 10x Genomics Chromium Single-Cell 3.1 according to the manufacturer’s instructions. The libraries from control, *Reverb*-hDKO, and *Reverb/Scap*-hDKO livers were constructed according to the 10x Genomics Chromium Single-Cell 3.1 manufacturer’s instructions and sequenced with NovaSeq 6000.

### Single-cell RNA-Seq data analysis.

Cell Ranger (3.0.2) software (10x Genomics) was run on the raw data using mm10. Output from Cell Ranger was loaded into R package Seurat (version 3.0) (53). Unique sequencing reads for each gene were normalized to total unique molecular identifiers (UMIs) in each cell to obtain normalized UMI values. Unsupervised clustering was applied after aligning the top 20 dimensions resulting from the principal component analysis (PCA) space using a resolution of 0.5. The identity for each cluster was assigned on the basis of prior knowledge of marker genes. The DEGs in each cluster were defined using a *P* value cutoff of less than 0.01 and a fold change of greater than 1.2. The uniform manifold approximation and projection (UMAP) plots, violin plots, Venn diagrams, and heatmaps were generated in R.

### RNA extraction and quantitative PCR.

To determine REV-ERB target genes while minimizing the batch effects, we collected livers, extracted RNA, and generated RNA-Seq libraries at the same time. At each time point, we alternated the sacrifice of control *Reverb*-hDKO, and *Reverb/Scap*-hTKO mice to further avoid bias. Total RNA was extracted from snap-frozen liver tissues using TRIzol reagent (Thermo Fisher Scientific) followed by the RNeasy Mini Kit (QIAGEN). RNA was reverse-transcribed using the High-Capacity cDNA Reverse Transcription Kit (Thermo Fisher Scientific). Quantitative PCR (qPCR) was performed with Power SYBR Green PCR Master Mix (Thermo Fisher Scientific) and a QuantStudio 6 Flex instrument (Applied Biosystems), and analysis was performed using the standard curve method. Gene expression was normalized to the mRNA level of the housekeeping gene *Arbp* and the minimum expression level of the gene in the samples.

### RNA-Seq.

Total RNA was extracted (see *RNA extraction*) from isolated hepatocytes and LMs. Purified dNase-treated total RNA (1 μg) from biological replicates was processed with a Ribo-Zero Magnetic rRNA removal kit (Illumina). RNA libraries were prepared using a TruSeq Stranded Total RNA Library Prep kit (Illumina, catalog 20020599) according to the manufacturer’s protocol.

### RNA-Seq data processing.

RNA-Seq reads were aligned to the University of California, Santa Cruz (UCSC) mouse genome mm9 genome browser using STAR (54). The tag directories were established, and normalized read counts were measured with RefSeq genes using Homer (55). The identification of rhythmic transcripts, enhancers, and metabolites followed the guidelines for genome-scale analysis of biological rhythms (56). The vector of time-ordered reads per kb per 10 million reads (RPKTM) values for each feature was duplicated and input into JTK_CYCLE (57) as an 8–time point to allow thorough identification of oscillation patterns starting at each different time point as previously described (11). Only genes with a maximum RPKTM value of greater than 5 identified in 2 or more samples at 4 time points were selected for the downstream analysis. Oscillating transcripts were defined as those with a JTK_CYCLE adjusted *P* value of less than 0.05, an oscillation amplitude (peak/trough) of greater than 1.5, and an oscillation period within the range of 21–24 hours. Enrichr (58) and PANTHER (59, 60) were used for pathway enrichment and gene ontology (GO) analysis.

### Fast-ATAC-Seq.

The process of fast-ATAC-Seq has been previously described (61). In brief, 50,000 isolated LMs were resuspended in 50 μL transposase mixture (25 μL 2× TD buffer, 2.5 μL tagment DNA enzyme (TDE1) (Illumina, 15027865), 0.5 μL 1% digitonin, and 22 μL nuclease-free water) (Illumina, catalog 15027865). Transposition reactions were incubated at 37°C for 30 minutes in an Eppendorf Thermo Mixer with agitation at 300 rpm. Transposed DNA was purified using the QIAGEN MinElute Reaction Cleanup kit (catalog 28204), and purified DNA was eluted in 10 μL elution buffer (10 mM Tris-HCl, pH 8). Transposed fragments were amplified and purified as described previously (62). Libraries were quantified using KAPA Library Quantification Kits (Roche, KK4873). All fast-ATAC libraries were sequenced using paired-end, dual-index sequencing on an Illumina NextSeq 500 instrument.

### ATAC-Seq data processing.

The ATAC-Seq analysis pipeline developed by Anshul Kundaje (https://github.com/kundajelab/atac_dnase_pipelines) was applied. For each sample, adapters were trimmed and aligned to the mouse genome mm9 with Bowtie. The aligned BAM files of biological replicates were then merged and subjected to peak calling of open chromatin regions. The tag directories were established, and normalized read counts were measured using Homer (55).

### Diurnal rhythmic enhancer identification.

To identify diurnal rhythmic enhancers, we quantified intergenic eRNA expression as previously described (24). We first determined the intergenic enhancer region by filtering out those chromatin opening regions that overlapped with known coding regions and long noncoding RNAs (lncRNAs) (with a 1 kb extension from both the transcription start site and transcription end site). Then, reads that mapped to ±500 bp of the eRNA locus center were considered and further normalized to RPKTM using Homer (55). Only those loci identified in 2 or more samples in LMs from control or *Reverb*-DKO livers were selected for JTK_CYCLE analysis (57). Oscillating enhancers were defined as those with a JTK_CYCLE–adjusted *P* value of less than 0.06, a maximum RPKTM value of greater than 0.2, an oscillation amplitude (peak/trough) of greater than 1.5, and an oscillation period within the range of 21–24 hours.

### Motif mining and IMAGE analysis.

To identify TFs enriched in the loci of rhythmic enhancers, IMAGE analysis (63) was applied to integrate the signals from both rhythmic enhancers and transcripts. Motif mining was performed in each eRNA and transcript phase group using out-of-phase eRNAs and transcripts as a background. Next, the transcription activity of the identified motifs and the mean expression of their putative target genes were determined. The phase-specific regulatory predicted TFs were selected according to the following criteria: (a) the phase of TF transcription activity should match the phase of the mean expression of its putative target genes and (b) the rhythmic expression pattern was validated in the rhythmic transcriptome from LMs.

### Ligand receptor interaction analysis.

To predict which ligands produced by hepatocytes regulate the rhythmicity remodeling in LMs, R package NicheNet (available at GitHub: https://github.com/saeyslab/nichenetr) (28, 64) was applied to rhythmic transcriptomes from isolated hepatocytes and LMs. The circle plots were generated via R package circlize (65). The impact of REV-ERB in hepatocytes on the expression of the top predicated ligands was determined by the rhythmic transcriptome from hepatocytes, and the expression of receptors was determined by the rhythmic transcriptome from LMs. To validate the predicted ligand-receptor interaction, the immortalized KC line (MilliporeSigma, SCC119) was treated with 20 ng/mL recombinant PDGF (Gibco, Thermo Fisher Scientific, PHG0045) for 24 hours, and the expression of its target gene *Jund* was determined by qPCR.

### Western blotting.

An equivalent protein amount (30 μg) was loaded and separated by 10% SDS-PAGE gels and then transferred onto PVDF Immobilon-P Membranes (MilliporeSigma, IPVH00010, ISEQ00010). The membranes were then incubated at 4°C overnight with the anti–PDGF-D polyclonal antibody (1:1,000; MilliporeSigma, catalog 40-2100) or anti–β-actin antibody (1:10,000; Cell Signaling Technology, catalog 4967). After washing 3 times with PBS containing 10% Tween-20 (PBST), the membranes were incubated with HRP-linked goat anti–rabbit IgG (1:3,000; Cell Signaling Technology, catalog 7074). Pierce ECL Western Blotting Substrate (Thermo Fisher Scientific, catalog 32209) was used for visualization.

### Lipid measurement by LC-MS.

Hepatocytes were extracted with –20°C isopropanol (100 μL per ~10^6^ cells), vortexed, and centrifuged at 16,000*g* for 10 minutes at 4°C. The supernatant was collected for liquid chromatography–mass spectrometry (LC-MS) analysis. A Q-Exactive Plus Quadrupole-Orbitrap mass spectrometer (Thermo Fisher Scientific) operating in positive ion mode was coupled via electrospray ionization and used to scan from *m/z* 290 to 1,200 at 1 Hz and 140,000 resolution. LC separation was done on an Atlantis T3 Column (2.1 mm × 150 mm, 3 μm particle size, 100 Å pore size; Waters) using a gradient of solvent A (1 mM ammonium acetate, 35 mM acetic acid in 90:10 water/methanol) and solvent B (1 mM ammonium acetate, 35 mM acetic acid in 98:2 isopropanol/methanol). The flow rate was 150 μL/min. The LC gradient was as follows: 0 minutes, 25% B; 2 minutes, 25% B; 5.5 minutes, 65% B; 12.5 minutes, 100% B; 16.5 minutes, 100% B; 17 minutes, 25% B; 30 minutes, 25% B. The autosampler temperature was 4°C, and the injection volume was 3 μL. Data were analyzed using the Compound Discoverer (Thermo Fisher Scientific) and Maven software.

### Metabolomics by LC-MS.

LMs were extracted with ice-cold acetonitrile/methanol/water (40:40:20) solution (100 μL per ~10^5^ cells). Following vortexing and centrifugation at 16,000*g* for 10 minutes at 4°C, 70 μL supernatant was loaded into MS vials. Metabolites were analyzed by quadrupole-orbitrap mass spectrometer coupled to hydrophilic interaction liquid chromatography (HILIC) via electrospray ionization. LC separation was done on an Xbridge BEH amide column (2.1 mm × 150 mm, 2.5 μm particle size, 130 Å pore size; Waters) at 25°C using a gradient of solvent A (5% acetonitrile in water with 20 mM ammonium acetate and 20 mM ammonium hydroxide) and solvent B (100% acetonitrile). The flow rate was 150 μL/min. The LC gradient was as follows: 0 minutes, 90% B; 2 minutes, 90% B; 3 minutes, 75% B; 7 minutes, 75% B; 8 minutes, 70% B; 9 minutes, 70% B; 10 minutes, 50% B; 12 minutes, 50% B; 13 minutes, 25% B; 14 minutes, 20% B; 15 minutes, 20% B; 16 minutes, 0% B; 20.5 minutes, 0% B; 21 minutes, 90% B; 25 minutes, 90% B. The autosampler temperature was set at 4°C, and the injection volume of the sample was 3 μL. MS data were acquired in negative and positive ion modes with a full-scan mode from *m/z* 70–830 and 140,000 resolution. Data were analyzed using the Compound Discoverer and Maven software.

### Diurnal rhythmic lipidomics and metabolomics.

Since other normalization methods by tissue mass or protein content are not accurate using 10,000–30,000 cells, quantile normalization was used to make the distributions the same across samples. Quantile normalization was performed using R package preprocessCore and was based on the concept of a quantile-quantile plot extended to *n* dimensions (66, 67). No special allowances were made for outliers. After quantile normalization, the rhythmic lipids and metabolites were determined by JTK_CYCLE analysis (57). Oscillating metabolites were defined as those with a JTK_CYCLE adjusted *P* value of less than 0.05, an oscillation amplitude (peak/trough) of greater than 1.5, and an oscillation period within the range of 21–24 hours. To determine the enriched pathway of metabolites in LMs, MetaboAnalyst 4.0 (68) was applied using an adjusted *P* value of less than 0.05 as the cutoff.

### Statistics.

R and Excel were used for graphing and statistical tests. Data represent the mean ± SEM, and statistical significance was determined by 2-tailed, 2-tailed *t* test, with a *P* value of less than 0.05 considered significant unless otherwise stated in the figure legends.

### Study approval.

All mouse care and use procedures were approved by the IACUC of the University of Pennsylvania and Baylor College of Medicine and were in accordance with NIH guidelines.

### Data availability.

The scRNA-Seq, RNA-Seq, and ATAC-Seq data reported in this study were deposited in the NCBI’s Gene Expression Omnibus (GEO) database (GEO GSE206319).

## Author contributions

DG and MAL conceptualized the study, interpreted data, and wrote the manuscript, which was revised and approved by all authors. DG and DS performed single-cell RNA-Seq, RNA-Seq, and ATAC-Seq analyses. DG, YC, PL, and C Jiang performed bioinformatics analysis. HB and C Jang. designed and performed lipidomics and metabolomics. CML, TMT, Y Xiong, KZ, WH, and BC assisted with animal husbandry and immortalized KCs. DG, D Zhou, and Y Xiao performed single-cell RNA-Seq, RNA-Seq, and ATAC-Seq analyses.

## Supplementary Material

Supplemental data

Supplemental table 1

Supplemental table 2

Supplemental table 3

Supplemental table 4

## Version 1 04/17/2023

Electronic publication
