# Supplementary material for: Hepatocyte SREBP signaling mediates clock communication within the liver
Source: J Clin Invest. 2023 Apr 17;133(8):e163018. doi: 10.1172/JCI163018 (PMC10104893; doi:10.1172/JCI163018)
Supplement: Supplemental data [file jci-133-163018-s095.pdf]

Figure S1

A

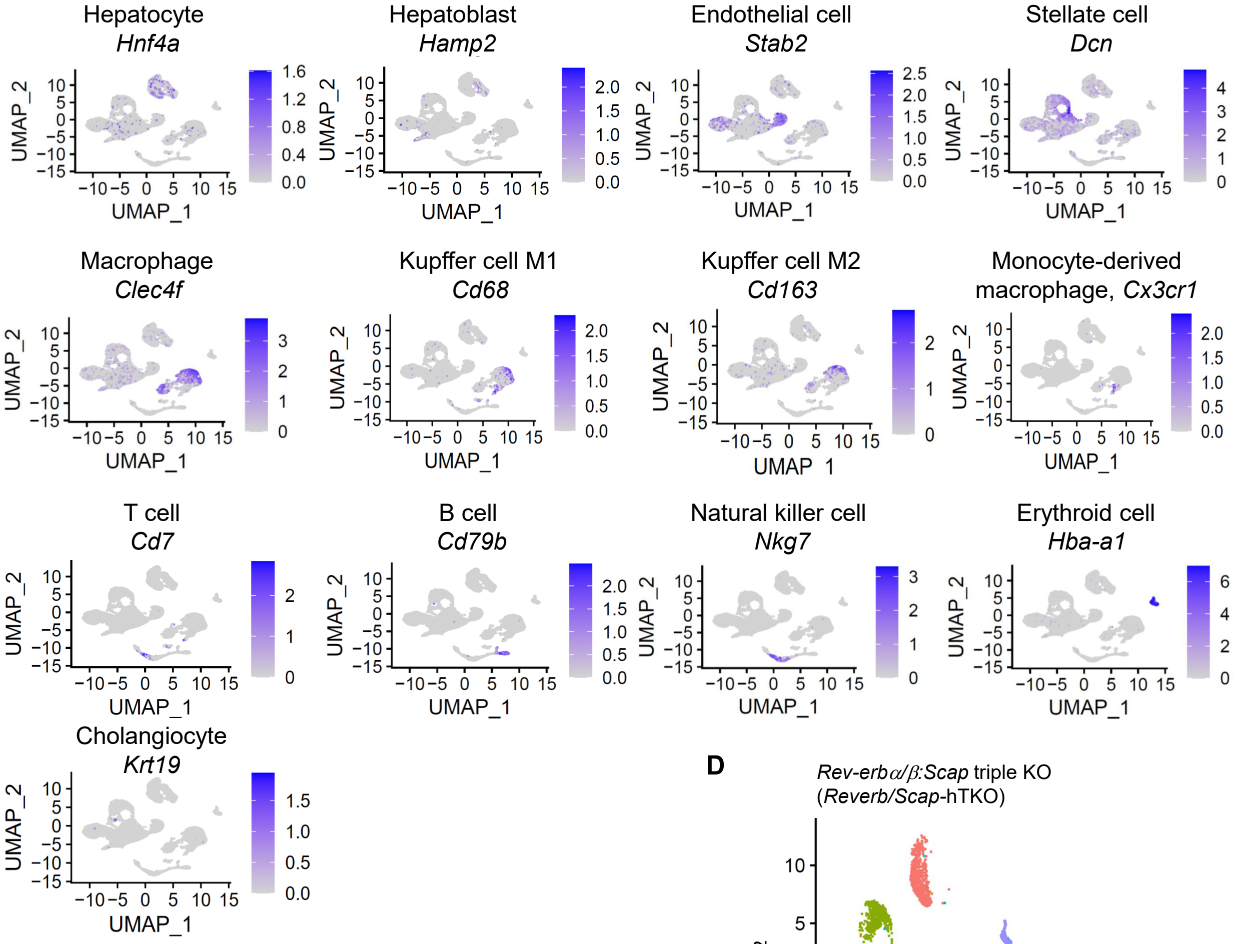

B

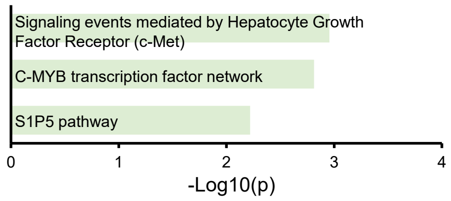

C

Cell	Control	*Reverb*-hDKO	*Reverb/Scap*-hTKO
Endothelial cell1 (EC1)	14.09	12.40	11.15
Endothelial cell2 (EC2)	13.63	9.70	12.41
Endothelial cell3 (EC3)	6.93	6.14	4.97
Kupffer cell M1 (KCM1)	12.42	11.55	14.38
Kupffer cell M2 (KCM2)	5.61	6.14	6.31
Monocyte-derived macrophages (MoM)	1.18	2.15	2.93
Stellate cell1 (SC1)	11.00	11.51	11.90
Stellate cell2 (SC2)	2.44	2.50	3.06
B cell (B)	3.13	2.08	2.29
T cell (T)	0.90	1.44	0.76
Natural killer cells (NK)	3.99	3.05	2.97
Erythroid cell (Erythroid)	1.00	1.10	4.27
Cholangiocyte (Chol)	0.94	1.56	1.27

D

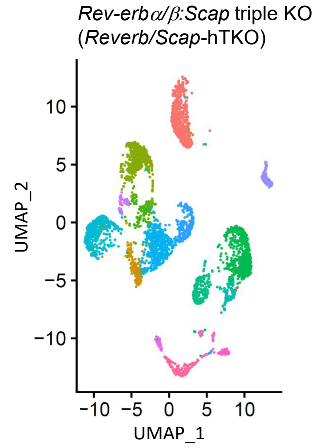

E

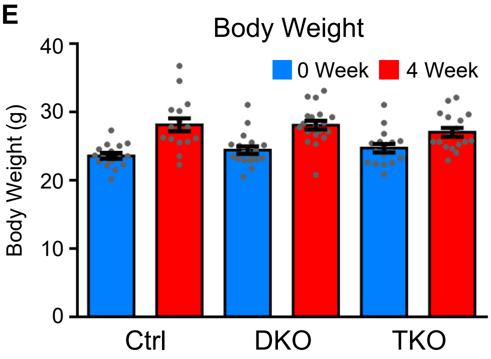

Figure S1. SREBP signaling is required for intraorgan communication from *Reverb*-hDKO hepatocytes. (**A**) UMAP plots displaying the expression of representative marker genes. (**B**) Pathways that were enriched (p < 0.01) in differentially expressed transcripts from hepatic stellate cells upon *Reverb*-hDKO. (**C**) Percentage of indicated cells in livers from 4-week HFHSD-fed control, *Reverb*-hDKO, and *Reverb/Scap*-hTKO mice. (**D**) UMAP visualization of cell clusters in livers from 4-week high fat high sucrose-fed *Reverb/Scap*-hTKO mice. (**E**) Body weight of each genetic model before and after 4-week HFHSD-feeding (n= 4-5).

Figure S2

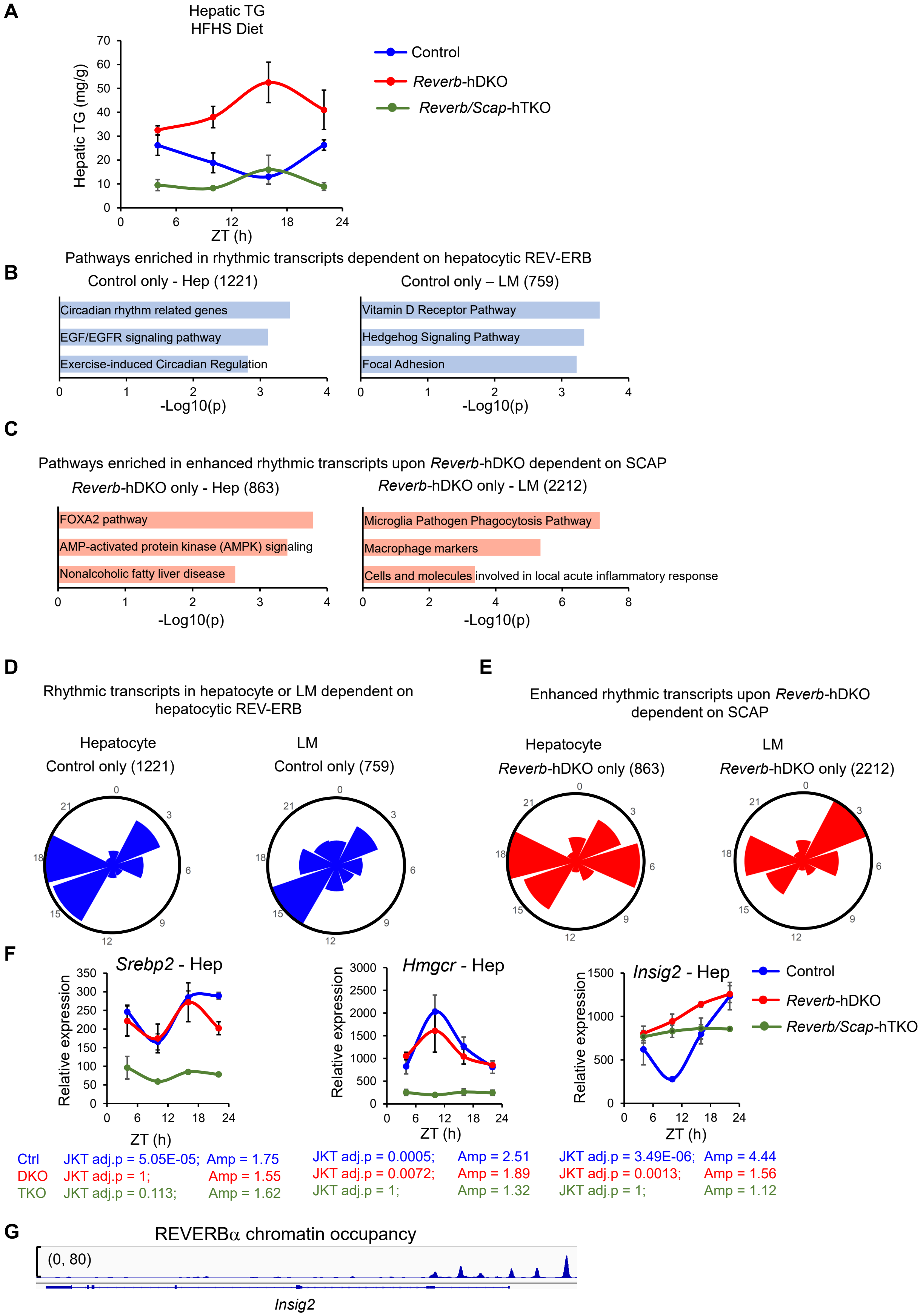

Figure S2. SREBP signaling is required for the rhythmic transcriptomic remodeling in LM upon *Reverb*-hDKO. (**A**) Hepatic TG measurements in HFHS-fed control, *Reverb*-hDKO, and *Reverb/Scap*-hTKO livers. Data are presented as mean ±SEM. (n = 3-4 per time point). (**B** and **C**) Pathways that were enriched (p < 0.01) in rhythmic transcripts dependent on hepatocytic REV-ERB (**B**) and enhanced rhythmic transcripts upon *Reverb*-hDKO dependent on SCAP (**C**). (**D**) Phase analysis of rhythmic transcripts in hepatocytes or LMs dependent on hepatocytic REV-ERB (**E**) Phase analysis of enhanced rhythmic transcripts upon *Reverb*-hDKO dependent on SCAP. (**F**) Relative expression of *Srebp2, Hmgcr, and Insig2* in hepatocytes. Data are presented as mean ±SEM. (n = 3 per time point). (**G**) Genome browser tracks of the Insig2 gene body and enhancer locus highlighting ChIP-seq data (PMID: 26044300) using anti-REV-ERBα antibody.

Figure S3

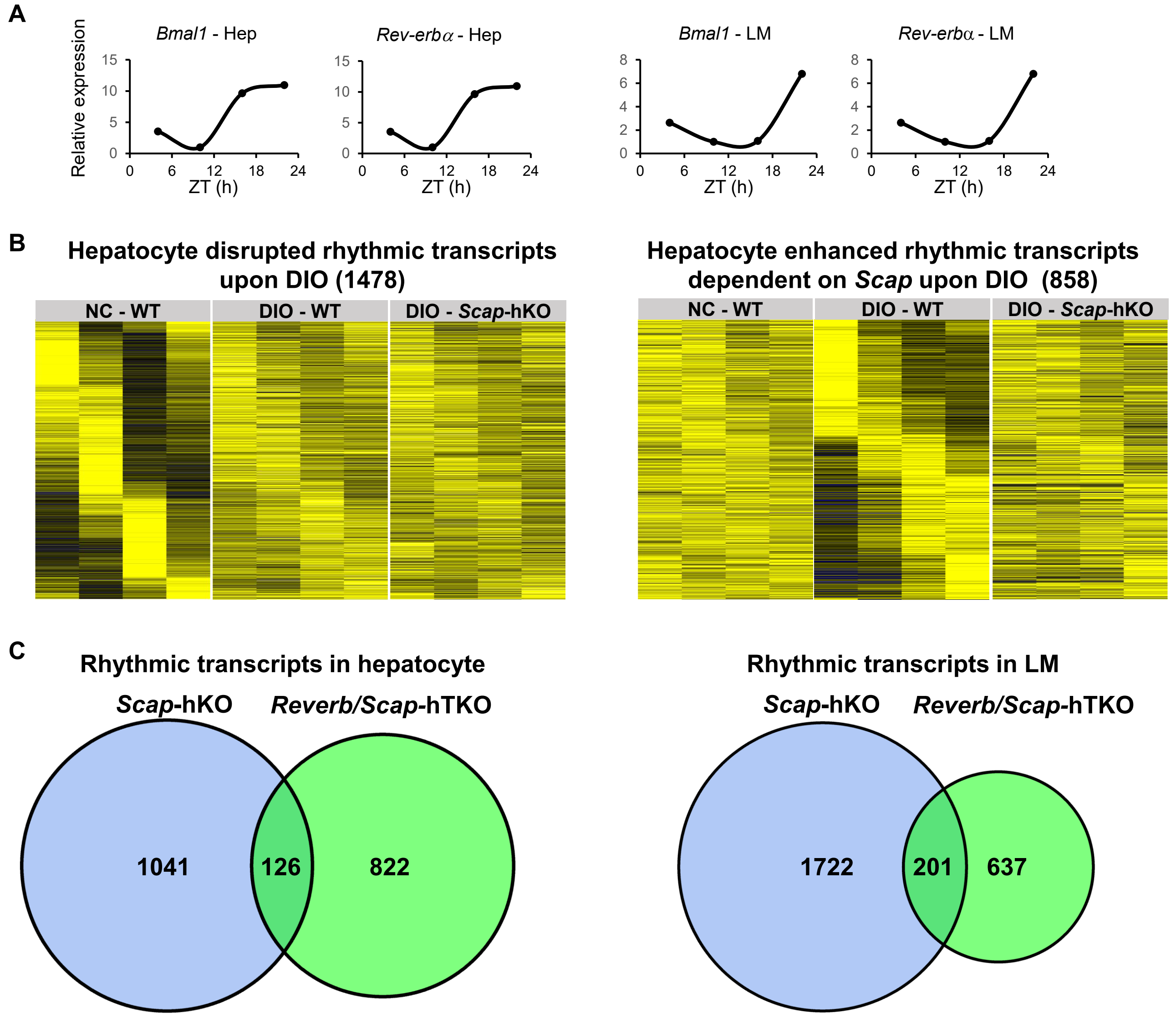

Figure S3. A majority of rhythmic transcript in *Reverb/Scap*-hTKO is different from *Scap*-hTKO. (**A**) Relative expression of core clock genes in hepatocytes and LM from *Scap*-hKO livers. Data are presented as mean ±SEM. (n = 3 per time point). (**B**) Heatmap of rhythmic transcripts in hepatocytes or LMs in livers from NC, DIO, and DIO-*Scap*-hKO mice. (**C**) Number of rhythmic genes in hepatocyte and LM from *Scap*-hKO and *Reverb/Scap*-hTKO livers.

Figure S4

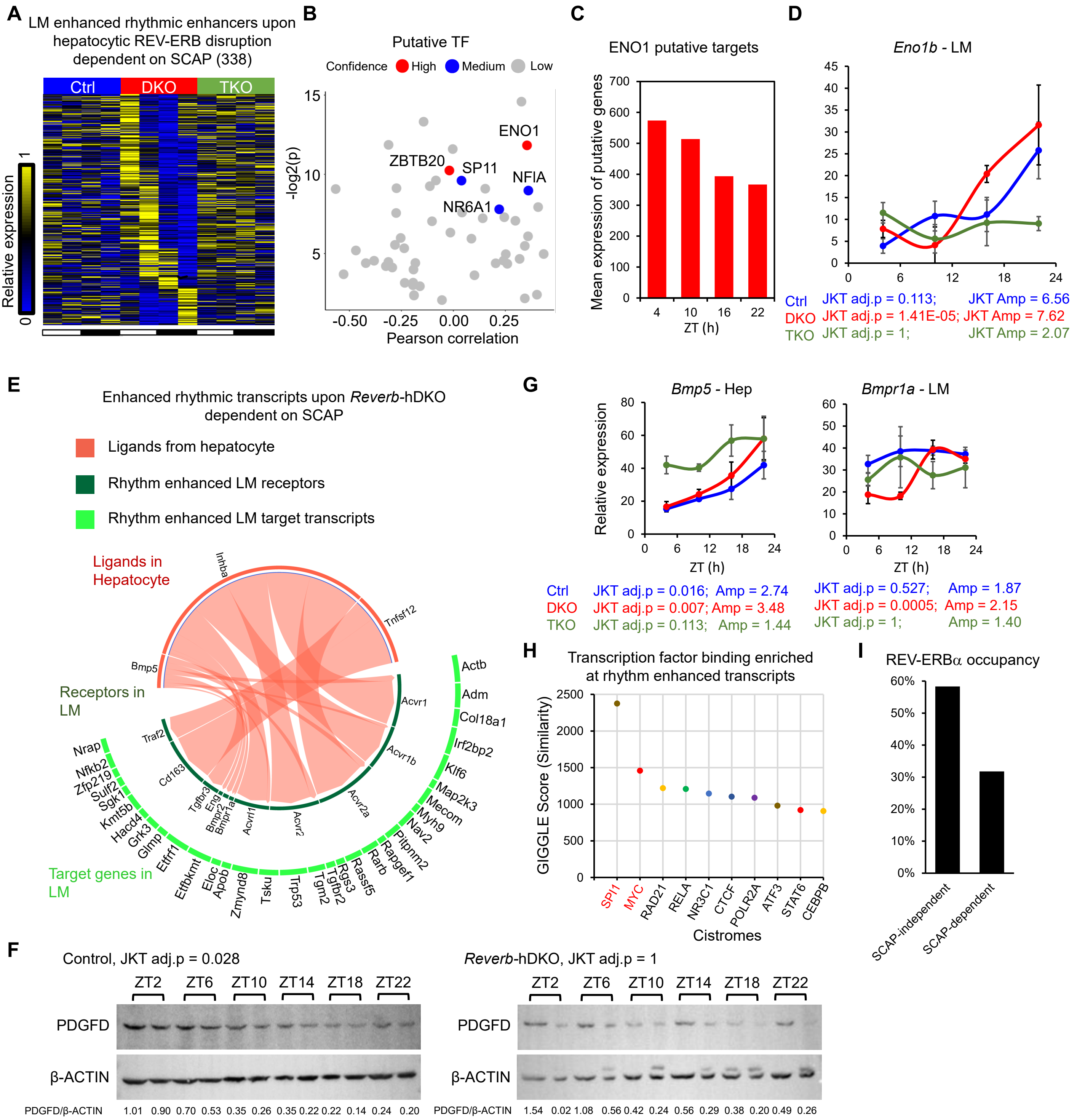

Figure S4. Ligands from hepatocytes reprogram REV-ERB-dependent oscillating enhancers in LMs. (A and B) Heatmap (A) and IMAGE analysis (B) of enhanced rhythmic enhancers in LM upon hepatocytic REV-ERB disruption dependent on SCAP. (C) Mean expression of putative target genes of ENO1 in isolated LMs from control liver. (D) Relative expression of *Eno1* of LMs in livers from 4-week high fat high sucrose-fed control, *Reverb*-hDKO, and *Reverb/Scap*-hTKO mice. Data are presented as mean ± SEM. (n = 3 per time point). (E) Ligand-receptor pair analysis. Circle plots showing links between predicted ligands from hepatocytes (tomato) with their associated receptors from LM (blue) associated with rhythmic transcripts in LM dependent on hepatocytic REV-ERB potentially targeted by the ligand-receptors pairs. (F) PDGFD protein levels in control and *Reverb*-hDKO livers. (G) Relative expression of *Bmp5* in hepatocytes and *Bmpr1a* in LMs. Data are presented as mean ±SEM. (n = 3 per time point). (H) TF binding similarity screening on rhythm enhanced transcript upon *Reverb*-hDKO and dependent of SCAP. (I) Percentages of SCAP-independent and SCAP-dependent rhythmic genes in *Reverb*-hDKO whose gene body genomic locus contains REV-BER binding sites.

Figure S5

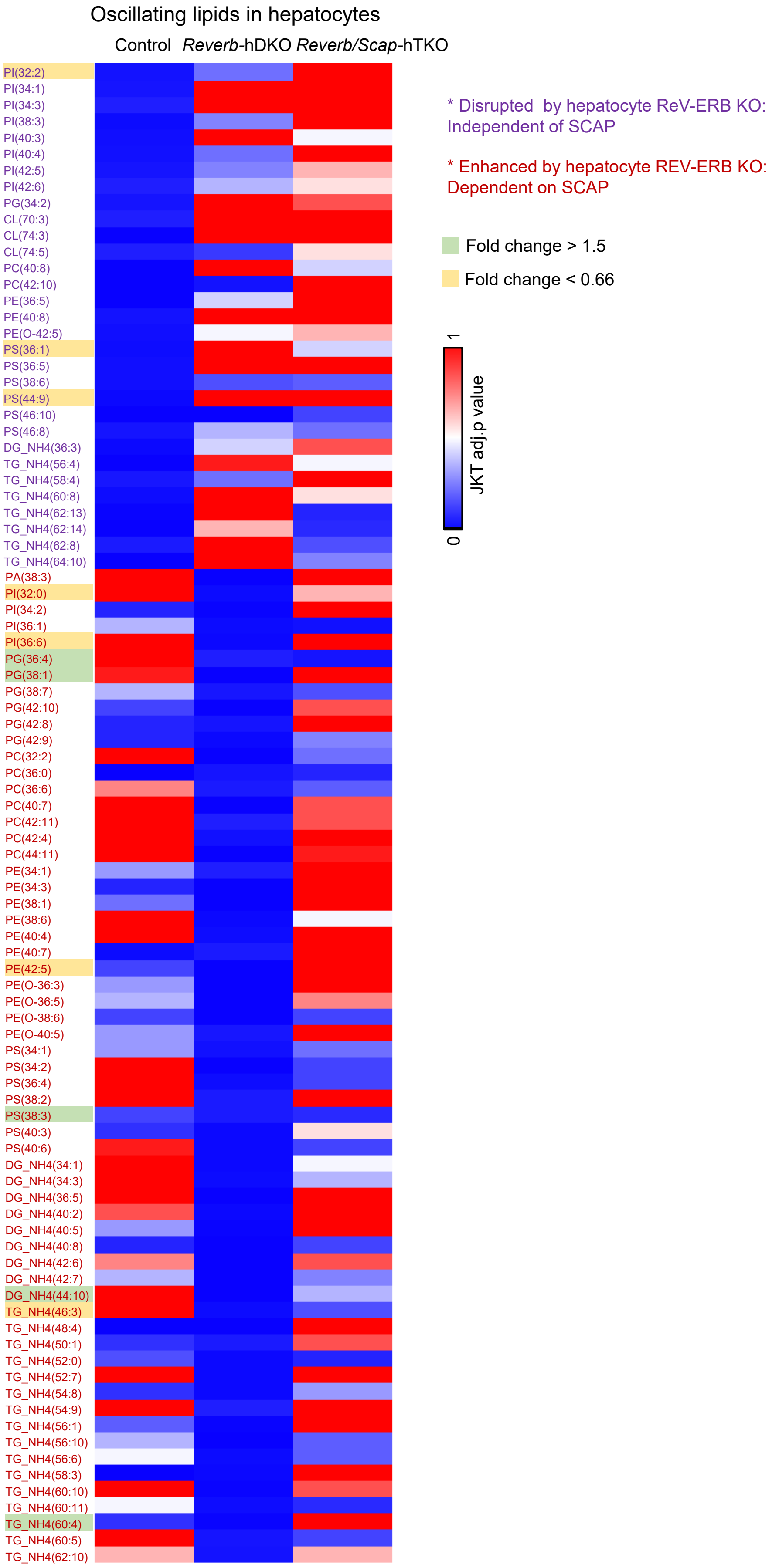

Figure S5. LM diurnal rhythms controlled by SCAP-dependent lipid metabolism in REV-ERB-depleted hepatocytes. Heatmap of rhythmic lipids in hepatocytes upon hepatocytic REV-ERB disruption independent and dependent on SCAP. PI, phosphatidylinositol; PG phosphatidylglycerol; CL, cardiolipin; PC, phosphatidylcholine; PE, phosphatidylethanolamine; PS, phosphatidylserine; DAG, diacylglycerol; TAG, triacylglycerol. The rhythms of lipids independent on SCAP upon hepatocytic REV-ERB disruption were labeled as purple, and SCAP-dependent lipids were labeled as violet. The lipids whose abundance was increased upon hepatocytic REV-ERB disruption have a light-green background, and the lipids with decreased abundance have a light-yellow background.
